# Peeling back the layers of immunogenicity in Cas9-based genomic medicine

**DOI:** 10.1016/j.ymthe.2025.06.045

**Published:** 2025-07-01

**Authors:** Virpi Stigzelius, Anna Lina Cavallo, Rakesh Kantilal Chandode, Roberto Nitsch

**Affiliations:** 1 Cell Therapy Safety, Clinical and Pharmacological Safety Sciences, AstraZeneca R&D, 431 83 Mölndal, Sweden; 2 Drug Research Program, Faculty of Pharmacy, University of Helsinki, Helsinki 00014, Finland; 3 Immune Analytics & CGT Dx Bioanalysis, Clinical Pharmacology and Safety Sciences, AstraZeneca R&D, 431 83 Mölndal, Sweden; 4 Immunology Safety, Safety Sciences, Clinical Pharmacology and Safety Sciences, AstraZeneca R&D, 431 83 Mölndal, Sweden

**Keywords:** genomic medicine, CRISPR-Cas9, immunogenicity, host immune response, genetic diseases

## Abstract

The CRISPR-Cas9 genome editing system is rewriting the treatment of genetic disorders, offering unprecedented potential for detrimental and previously untreatable diseases. As this technology advances toward wider utilization in clinical applications, the immunogenicity of Cas9 nuclease has emerged as a potential challenge for *in vivo* therapies. Immune recognition of CRISPR-Cas9 components can trigger both innate and adaptive responses. The complex interactions between Cas9, delivery vectors, and host immune reactivity play a crucial role in determining the safety and efficacy of CRISPR-based treatments. Recent advances in mitigating Cas9 immunogenicity include epitope engineering, optimized delivery systems, and nucleic acid modifications. These strategies, explored across various tissue contexts and delivery methods, show promise in enhancing the tolerability of CRISPR-based therapies. However, pre-existing immunity to Cas9 and the potential for long-term adaptive immune responses remain important considerations. Addressing these immunological challenges requires an integrated approach, combining insights from immunology with innovative engineering solutions. As the field progresses, overcoming Cas9 immunogenicity will be crucial for realizing the full therapeutic potential of the CRISPR-Cas9 system in diverse clinical applications.

## Graphical abstract

**Figure:**
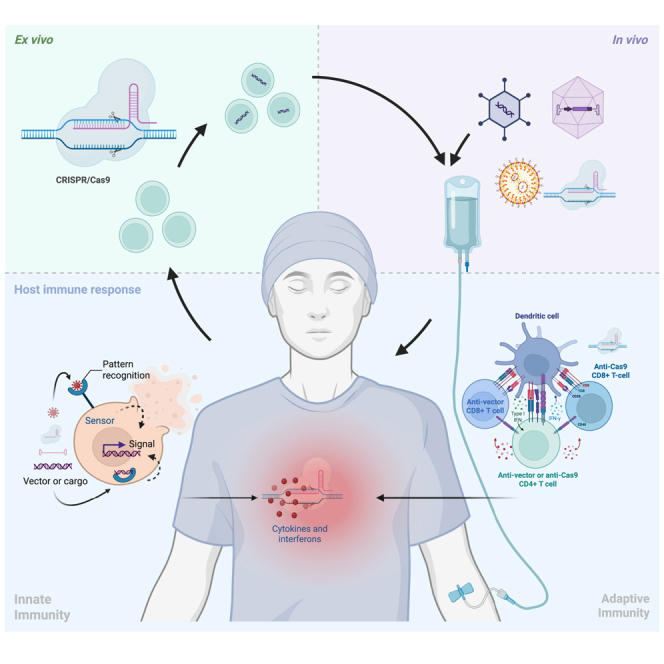

## Teaser

Nitsch and colleagues review potential immunological challenges related to CRISPR-Cas9-based therapies and current approaches to mitigate CRISPR-Cas9 component-mediated innate and adaptive responses. As pro-inflammatory host response may impair treatment efficacy and safety, understanding antigen-specific pre-existing immunity and host immune responses to treatment remain important considerations for clinical utilization of CRISPR-Cas9 system.

## Introduction

The immunogenicity of CRISPR-associated protein 9 (Cas9) presents a complex challenge in the development and application of gene editing therapies. The bacterial nuclease, essential for targeted genomic modifications, inherently possesses immunogenic potential due to its prokaryotic origin. The host immune response to Cas9 is influenced by an intricate interplay of variables, including the delivery method, duration of protein expression, target tissue immune environment, and intrinsic properties of the nuclease itself. As CRISPR-Cas9-based therapies advance through clinical trials, understanding and mitigating Cas9 immunogenicity has emerged as a critical consideration. Hence, we aim to shed light on the challenges and potential strategies to address Cas9 immunogenicity by reviewing various factors that impact Cas9 immunogenicity.

### The basic structure and function of Cas9 in genome editing

Cas9 is a bacterial nuclease^1^^,^^2^ utilized as a biotechnological tool in eukaryotic genome engineering due to its capacity to generate targeted double-stranded DNA (dsDNA) breaks when complexed with a target site-recognizing single-guide RNA (sgRNA).^3^^,^^4^^,^^5^

The Cas9 orthologs belong to the type II CRISPR-Cas antiviral protein family,^6^ originating from diverse bacterial strains^7^ such as *Streptococcus pyogenes* (SpCas9)^5^^,^^8^ and *Staphylococcus aureus* (SaCas9).^9^^,^^10^ Structurally, Cas9 is divided into two main lobes: the target recognition lobe and the nuclease lobe^8^ with distinct features.^3^^,^^4^^,^^5^^,^^8^ Functionally, the Cas9 protein forms a ribonucleoprotein (RNP) complex with the guide RNA (gRNA) and screens the dsDNA for its target cleavage site. The cleavage process is highly regulated and relies on the interaction of Cas9 with an ortholog-specific protospacer-adjacent motif (PAM) sequence (e.g., 5′-NGG-3′ and 5′-NNGRRT-3′ for SpCas9^11^ and SaCas9,^10^ respectively) recognition.

**Figure 1:**
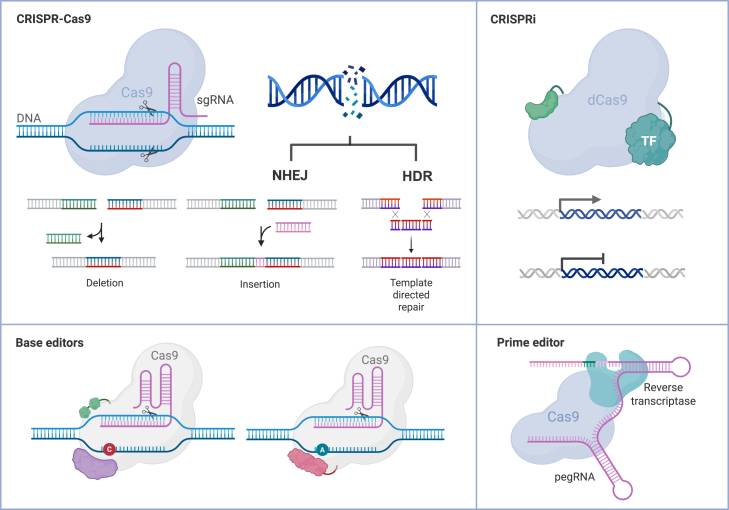
Cas9 is an essential element in the CRISPR-Cas9-based genome editor toolbox The CRISPR-Cas9 system is a gene-editing technology that utilizes a guide RNA to direct the Cas9 nuclease to a specific genomic location, where it introduces a double-strand break near a PAM site. Mutation generation occurs through two primary pathways: (1) NHEJ, which can result in insertions or deletions and (2) HDR, which allows precise editing by using a donor template for repair. In addition, gene expression can be regulated without generating double-stranded breaks. CRISPR-Cas9 epigenome or gene expression regulators, such as CRISPRa or CRISPRi are genome editor tools containing catalytically inactive dCas9 and a gene expression regulator unit for activation or repression of target genes, respectively. Cytosine and adenine base editors are engineered CRISPR-proteins fused together with cytosine or adenine deaminases for targeted base changes from C to T or A to G, respectively, through SSB generation (nickase). Prime editors are CRISPR-Cas9-based tools that enable a pegRNA template-based change introduction to the genome through engineered nickase fused with reverse transcriptase, directed by pegRNA. CRISPR-Cas9, clustered regularly interspaced short palindromic repeats/CRISPR-associated protein 9; PAM, protospacer adjacent motif; NHEJ, non-homologous end-joining; HDR, homology-directed repair; CRISPRi, CRISPR interference; dCas9, dead Cas9; pegRNA, prime editing guide RNA; SSB, single-strand break.

After the DNA cleavage, the introduction of changes to target genes relies on cells’ intrinsic DNA damage repair (DDR) processes (Figure 1). Upon a DNA strand cleavage, whether naturally occurring or generated through targeted gene editing, DNA damage sensor proteins^12^ complex with the severed DNA strands to promote replication stress and DNA repair pathway activation^13^^,^^14^ through mediator proteins, such as ataxia telangiectasia mutated (ATM) protein kinase.^12^ The two main dsDNA break repair processes include the “error-prone” non-homologous end joining (NHEJ), and a template-based sequence-restoring homology-directed repair (HDR). Moreover, cells can utilize a variety of minor DDR pathways (e.g., base excision repair and microhomology-mediated repair) for maintenance of genomic stability upon detection of DNA damage. In genome editing, DDR pathways and the heterogeneity in their utilization play a dual role, being both fundamental to the editing outcome and a challenge for targeted gene correction.

Substantial efforts to improve the utilization of Cas9-based genome engineering have led to an expanding toolbox of genome editors. These tools include engineered variants of the canonical nuclease such as: (1) PAM-relaxed^15^ Cas9 nucleases designed to circumvent the ortholog-specific PAM dependency^16^ to accommodate a broader range of genomic targets, (2) high-fidelity SpCas9^17^^,^^18^ to reduce undesired off-target cleavage and improve nuclease target specificity,^19^^,^^20^^,^^21^^,^^22^^,^^23^^,^^24^^,^^25^^,^^26^ and (3) Cas9 nickases^4^^,^^5^^,^^9^ enabling generation of ssDNA breaks to promote base excision and HDR repair processes for targeted gene correction. Furthermore, engineered fusion Cas9 nucleases such as base and prime editors^27^^,^^28^ have been generated by combining a Cas9 variant with an additional functional unit (e.g., reverse transcriptase) to promote controlled introduction of nucleotide-scale changes in the target gene. Similar approaches have been used for targeted regulation of gene expression without dsDNA cleavage^29^ by a combination of a catalytically deactivated Cas9 (dCas9) variant^30^ with epigenome or transcription modulator unit,^4^^,^^5^^,^^31^^,^^32^ enabling regulation of gene expression without permanent alterations of the host genome^33^ (Figure 1). Combined, the canonical Cas9 nucleases and their innovative successors offer means to address the underlying genetic causes for a variety of diseases. Nonetheless, despite the rapid evolution of genome editing tools with a multitude of Cas9-based genome editors in preclinical and clinical evaluation, the understanding of genome editor interaction with the host immune system is lagging.

### Activation of the host immune response

The immune system is a surveillance and defense mechanism that maintains homeostasis and protects the host from pathogen invasion. The system is divided into two main categories consisting of specialized cells, tissues, and organs: (1) the innate immune compartment, which provides a non-specific and rapidly activated first line of defense and (2) the adaptive immune compartment, which generates antigen-specific immunological memory.

**Figure 2:**
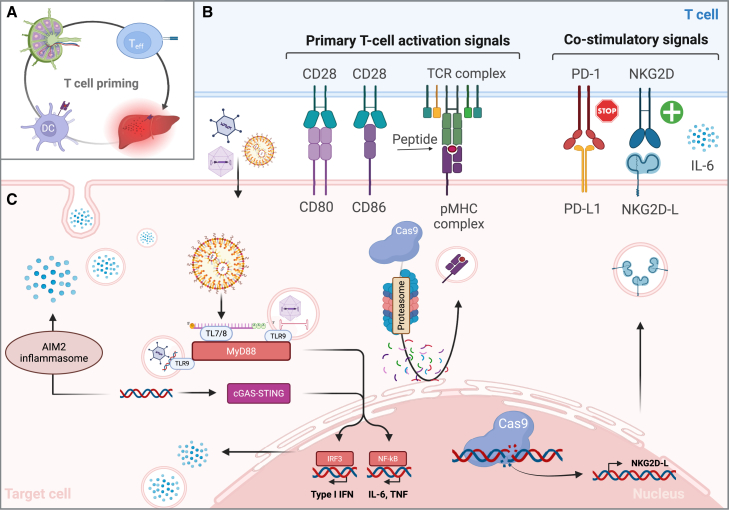
Development of antigen-specific cellular immune response Antigen-specific T cells response is generated by (A) antigen-presenting cells (e.g., DCs) during lymph nodal priming of naive T cells, which in turn, as activated T effector cells migrate to the affected tissues. (B) The cytolytic CD8+ T cells are activated based on two primary co-stimulatory signals. The primary activation signal is given by TCR-mediated recognition of the immunogenic antigen presented on the surface of the affected cells as pMHC complex. The secondary signal involves binding of co-stimulatory molecules, such as CD28 and CD80 or CD86, amplifying the activation signaling. Moreover, the T cell response can be regulated through additional signaling molecules, including localized cytokines, chemokines, and receptor-ligand interactions (e.g., PD-L1 or NKD2G-L upregulated by affected cells to promote tolerance or cytolytic activity, respectively). (C) During gene editing, the target cells are exposed to numerous pro-inflammatory factors, which may amplify the immunogenicity of the Cas9 protein, promoting Cas9 epitope-specific T cell priming and effector activity. These factors include intrinsic immunogenicity of the delivery vector system, nucleic acid sensing by the innate immune system, and inflammatory response to genotoxic stress. DC, dendritic cell; Teff, T effector cell; TCR, T cell receptor; AIM2, Interferon-inducible protein AIM2: pMHC, peptide-major histocompatibility complex; PD-1, programmed cell death rotein 1; NKG2D, natural killer group 2 member D; TLR, Toll-like receptor; MyD88, myeloid differentiation primary response 88; cGAS-STING, cyclic GMP-AMP synthase-stimulator of interferon genes; IRF, interferon regulatory factor; IFN, interferon.

Upon tissue damage or pathogen invasion, the innate immune response is activated through signaling cascades mediated by pattern recognition receptors (PRRs). During a process termed innate sensing, PRRs including Toll-like receptors (TLRs)^34^ and cyclic GMP-AMP synthase (cGAS)^34^^,^^35^ bind to danger- or pathogen-associated molecular patterns (DAMPs and PAMPs, respectively) such as mammalian DNA and bacterial components. DAMP or PAMP detection activates several immunological processes resulting in pro-inflammatory cytokine secretion, complement system activation, and recruitment of innate immune cells (e.g., macrophages and natural killer [NK] cells) and clearance of affected cells at the inflammation site.^36^ Furthermore, the innate immune compartment plays a vital role in the development of antigen-specific immunological memory (Figure 2). The initiation (i.e., priming) of an adaptive immune response begins at the affected tissue area where antigen-presenting cells (APCs) such as dendritic cells capture and process pathogen and cell fragments for antigen presentation to naive lymphocytes. The antigen presentation occurs through the major histocompatibility complex (MHC) membrane proteins, which are continuously shuffled to intracellular antigen-processing compartments for self and non-self-polypeptide loading. Based on their intrinsic antigenic features, a portion of polypeptides bind to the MHC, forming a peptide:MHC complex (pMHC). This complex is trafficked to the plasma membrane for extracellular antigen presentation to lymph nodal T and B cells,^37^^,^^38^ which in turn are activated by immunogenic peptides recognition through pMHC binding to T and B cell receptors (TCRs and BCRs, respectively), resulting in lymphocyte proliferation and differentiation into effector or antigen-specific memory cells.

Functionally, T cells are subcategorized into two main groups: cytolytic CD8+ T cells and mainly cytokine-secreting CD4+ T cells, primed and activated through MHC class I and II, respectively.^36^^,^^37^^,^^38^ The engagement between TCR and pMHC complexes provides the initial signal for T cell activation. Additionally, a secondary co-stimulatory signal through APC and T cell membrane proteins (e.g., CD80 and CD28, respectively) is required for T cell activation.^39^ Together, this main co-stimulatory signaling protects the host from autoreactivity (i.e., response against unaffected host cells) and mediates naive T cell differentiation into pro-inflammatory effector CD8+ and CD4+ T cells yielding protective immunity,^40^ including rare CD4+ T cell subpopulations with cytolytic capacity.^41^^,^^42^ Furthermore, depending on the local immune signaling context, additional co-stimulatory signals within the given immune environment can direct the development of antigen-specific host responses toward immunological tolerance,^43^^,^^44^^,^^45^^,^^46^^,^^47^^,^^48^^,^^49^ contributing to the generation of diverse antigen-specific immunological memory (Figure 2).

Additionally, during the primary infection, naive B cells are primed upon antigen encounter in the secondary lymphoid organs. The priming can occur through direct antigen binding to the BCR, or by APC-mediated antigen presentation combined with CD4+ T cell co-stimulatory cytokine (e.g., IL-4) signaling.^38^ Similarly to T cells, APC-mediated B cell activation requires a co-stimulatory receptor and ligand binding. Upon activation, naive B cells undergo clonal expansion and differentiation into antibody-producing plasma blasts with a limited lifespan and long-term plasma cells. These antibodies (e.g., IgG and IgM) neutralize pathogens and promote pathogen recognition by other immune system compartments. During the B cell priming and activation, an antigen-specific memory B cell population is generated in the lymph nodes, ensuring a rapid humoral response upon secondary infection. Moreover, upon antigen encounter, B cells can undergo a process called somatic hypermutation, introducing random mutations to the immunoglobulin gene variable region, resulting in antibodies with altered antigen binding affinity.^50^ This process selectively increases antigen specificity through antibody affinity maturation while also providing a source for cross-reactive low-affinity antibodies.

The MHC region is one of the most polymorphic areas of the vertebrate genome^51^ resulting in heterogeneity in disease susceptibility. In the human population, the MHC region encodes human leukocyte antigen (HLA) alleles with six main gene loci: HLA-A, HLA-B, and HLA-C or HLA-DP, HLA-DQ, and HLA-DR for MHC class I and II, respectively.^52^ This allelic variation generates diverse HLA haplotypes (i.e., combinations of the HLA gene variants) with different antigen-binding specificities, resulting in variable epitope recognition and broad adaptive immunity-mediated disease resistance.

Taken together, the immune system contains innate and adaptive immune compartments that together mediate host protection. The activation of the adaptive immune response is regulated through antigen presentation and co-stimulatory receptors, which depending on the signaling, can result in clearance or tolerance of the affected cells. The host immune system specificity is determined by an individual’s genetic and environmental exposure to antigens, resulting in heterogeneity in immunological reactivity. A diverse and broad immunity is vital for disease prevention and control. However, in a therapeutic context, the host immune response against the treatment modality can pose a significant obstacle to treatment administration and outcome.

### Gene therapy and host immune response

Gene therapies are advanced medical treatments that target genetic defects by mediating their effects through transfer of gene products or altering the function of dysfunctional genes. Traditional gene therapy involves *ex vivo* or *in vivo* treatment with a recombinant viral vector system and its transgene cargo,^53^ such as a gene construct for the factor VIII-clotting factor used in the treatment of a hereditary bleeding disorder, hemophilia A. Numerous gene therapies^54^^,^^55^^,^^56^^,^^57^^,^^58^^,^^59^^,^^60^^,^^61^ have shown clinical benefit for patients with otherwise poorly treatable diseases. However, the introduction of these complex therapeutic modalities to the host system is known to elicit pro-inflammatory anti-drug immune responses, potentially compromising treatment safety and efficacy.

The immunogenicity of gene therapy products is influenced by several factors, including manufacturing process-related impurities, the selection of delivery vector system, the transgene construct composition, and the immunogenicity of transgene product.^62^ When assessing the impact of pro-inflammatory response on treatment outcome, four key considerations should be taken into account. First, host immunity against gene therapy modalities may arise *de novo* in response to treatment administration^55^^,^^56^^,^^57^^,^^58^^,^^61^^,^^63^^,^^64^ or as a secondary memory response due to prior exposure to pathogens.^65^^,^^66^^,^^67^ Secondly, similarly to infusion of traditional therapeutic proteins,^68^ development of neutralizing and binding anti-drug antibodies against the treatment modality components (e.g., viral capsid proteins^63^^,^^69^) can reduce treatment bioavailability and efficacy, preventing sequential treatment unless otherwise managed. Thirdly, a cellular host response^70^^,^^71^^,^^72^^,^^73^ can result in clearance of treated cells expressing therapeutic modalities and transgene products due to the introduction of neoantigens,^67^^,^^69^^,^^74^^,^^75^^,^^76^ diminishing transgene stability and treatment efficacy. Finally, severe adverse events such as treatment-induced cardio and hepatotoxicity,^77^ including fatal reactions,^67^^,^^78^^,^^79^^,^^80^^,^^81^ may arise in response to gene therapy treatment administration.

In addition to the intrinsic features of therapeutic agents, several patient-specific factors play a role in activating immune responses against the treatment modalities. These include the route of administration and the target tissue immune context. For instance, localized treatment of an immune-privileged site (i.e., tissues and organs with anatomical or cellular adaptations that mitigate pro-inflammatory responses), such as the eye, is associated with a better immunological safety profile and less pronounced host immune response in most patients.^82^ On the other hand, in immunologically active tissues such as skeletal muscle,^83^ especially under inflammatory conditions, gene therapy can result in robust treatment-induced inflammatory reactions and loss of transgene expression.^74^^,^^75^ Moreover, the context of the immune environment and signaling directs the formation of host response, as observed through the complexity of immune surveillance in more tolerogenic tissues such as the liver.^84^ Notably, treatment-induced pro-inflammatory host immune responses have been reported in patients undergoing treatment approaches that target tolerogenic and compartmentalized organs,^85^ highlighting individualized diversity in immune responses. Taken together, the immunogenicity of gene therapy modalities, such as vector systems or their cargo, can result in anti-drug host immune responses. Activation of innate and subsequent adaptive host responses can lead to the elimination of targeted cells and trigger immune-related toxicity. Importantly, the type of immune response in relation to therapeutic modality and its delivery plays a central role in the clinical considerations. For instance, the clinical risks associated with therapeutic protein infusion^68^ typically induce an MHC class II-mediated host response (i.e., CD4+ T cell and B cell response), promoting neutralization and clearance of the therapeutic protein, impacting treatment efficacy and dosing. On the other hand, gene therapy approaches that rely on intracellular expression of the treatment modalities (i.e., viral vector-based therapies) promote class I-driven cytolytic (CD8+ T cell) response, increasing the risk for tissue damage. Hence, understanding treatment-related immunogenicity is critical for developing safe and effective treatments, particularly when utilizing components with known immunologically relevant properties.

## Cas9 immunogenicity—A multilayered question

The bacterial origin of Cas9 underlines the potential of the protein family members to be recognized as non-self by the mammalian immune system. However, the question of Cas9 immunogenicity is not straightforward and is affected by multiple variables. The host response to Cas9 variants, whether native or engineered, is influenced by factors relevant from traditional gene therapies (e.g., the delivery system, duration of protein expression, and tissue-specific immune context) as well as by novel factors such as immunogenicity of the genome editor and its DNA cleavage activity.

### Pre-existing immunity—Cas9 as a bacterial protein

The concern of pre-existing Cas9 immunity in humans was raised through the discovery of host antibodies in the human population against SpCas9 and SaCas9.^86^^,^^87^ The humoral response across the study-specific population cohorts varies, with detected prevalence of serum antibodies against SpCas9 ranging from 2.5%^86^ to 65%,^87^ and SaCas9 from 10%^86^ to 79%.^87^ This discrepancy highlights both individual variability in immunoreactivity and the need for the development of standardized screening approaches upon clinical monitoring. The naturally occurring humoral host response to Cas9 has since been observed in various healthy donor^86^^,^^87^ and patient populations.^88^^,^^89^^,^^90^ However, the potential cause, such as concurrent bacterial and bacteriophage infection^91^ or epitope cross-reactivity remains to be elucidated. Moreover, pre-existing SpCas9^92^ and SaCas9^93^^,^^94^ antibodies are reported in preclinical animal models. Some mammalian commensal bacterial strains such as *Staphylococcus aureus*^95^ exhibit a capacity for zoonotic infection, and while *Streptococcus pyogenes* is primarily observed in humans, sporadic colonization infectivity in animals may occur.^96^ Importantly, SpCas9 shares an 89% sequence homology with a Cas9 ortholog originating from *Streptococcus canis*,^97^ a strain commonly observed in canines, presenting a possibility of shared immunogenic epitopes and a potential explanation for the observations of anti-SpCas9 host antibodies in canine models. Hence, for an evaluation of genome editors’ immunological safety, it is recommendable to establish the baseline status of anti-Cas9 host reactivity upon treatment administration. However, unlike most therapeutic proteins, where humoral host response assessment is based on comparison with samples from individuals who have never received the treatment, the pathogenic origin of Cas9, combined with wide pre-exposure, presents a challenge in setting reliable response detection thresholds. Additionally, further studies are needed to understand potential cross-reactivity of immune cells to Cas9 orthologs, which will likely require the use of more sensitive and specific antibody detection methods compared with conventional enzyme-linked immunosorbent assays (ELISAs).

In addition to humoral responses, pre-existing Cas9 T cell immunity presents a potential challenge for *in vivo* genome editing. T cell immunity can be assessed, for example, on a functional level using peripheral blood mononuclear cell (PBMC) or splenocyte co-cultures and antigen recall assays that measure antigen recognition-induced T cell activation, commonly detected based on pro-inflammatory cytokine (e.g., interferon-γ) expression and secretion. Cas9-specific pro-inflammatory responses have been detected in both CD4+ and CD8+ T cells upon full-length protein or peptide stimulation.^86^^,^^98^^,^^99^^,^^100^ Early evidence of SaCas9-reactive CD4+ T cells in humans stems from flow cytometry-based analysis of intracellular pro-inflammatory cytokine responses ranging from 9.5% to 52% and from 14% to 53% in a donor population representing common North American HLA haplotypes.^101^ A combinatorial Cas9 epitope assessment with computational overlay of mass spectrometry-based immunopeptidome (i.e., pMHC-mediated antigenic peptide presentation) analysis and T cell activation assays revealed HLA-allele-specific differences for SaCas9 epitope reactivity, highlighting donor-specific variability. In complementary studies investigating SpCas9^99^^,^^100^ and SaCas9^98^ immunogenicity, both CD4+ and CD8+ T cell reactivity have been observed in the human population to varying degrees, based on ELISpot and flow cytometric detection. Moreover, pro-inflammatory T cell response has been reported to coincide with a Cas9-specific immunosuppressive regulatory T cell response,^100^ indicating both Cas9 tolerance and pre-existing T cell immunity. Of importance, the majority of the effector T cell population resides in both lymphoid and non-lymphoid tissues.^102^ While the circulatory T cell populations, commonly used for host immune reactivity screening, provide essential insights into immunoreactivity, they represent a minority of total T cells and, therefore, introduce an intrinsic bias to the measurement, failing to capture tissue-resident T cell responses.

Together, these studies highlight pre-existing T cell immunity to Cas9 in humans as well as the technical complexity in detecting T cell response. Notably, the understanding of T cell-mediated responses highlights the activation process, which does not directly translate to cytolytic activity. Cas9-specific T cell-mediated target cell rejection remains speculative, warranting further exploration.

### Treatment-induced immunity—Cas9 as a therapeutic protein

Similarly to traditional gene therapies, two main treatment administration approaches—*ex vivo* and *in vivo*—are utilized within the emerging field of therapeutic gene editing. Both administration methods can induce a *de novo* pro-inflammatory host response and are impacted by treatment approach and modality. The advancement of genome editing technologies is accompanied by an increasing diversity in their delivery formats (e.g., DNA, RNA, or protein with various vector systems), introducing an additional level of complexity in therapy-induced host response evaluation. The first CRISPR-Cas9-based therapies have been approved for the treatment of severe sickle cell anemia^103^ and transfusion-dependent β-thalassemia.^104^ Additionally, a wide range of *ex vivo* and *in vivo* approaches are currently in clinical evaluation for numerous indications,^105^ ranging from autoimmune disorders^106^ to cancer.^107^

#### Host response to *ex vivo* Cas9-based genomic medicine

The approved treatment, exagamglogene autotemcel (exa-cel, marketed by name CASGEVY), utilizes *ex vivo* SpCas9-RNP-induced silencing of erythroid lineage-specific *BCL11a* in autologous CD34+ hematopoietic stem and progenitor cells (HSPCs) to restore expression of fetal hemoglobin.^103^^,^^104^ Prior to receiving the single-dose infusion, patients undergo HSPC mobilization and apheresis for the production of autologous cells, followed by a 4-day myeloablative conditioning for the cell product transplantation. The treatment is administered over a 7-day administration window, based on patient tolerance. Results from phase 3 clinical trials have demonstrated significant improvement in disease-related symptoms (e.g., vaso-occlusive episodes), reducing the need for patient hospitalization, along with a safety profile similar to that of a traditional autologous stem cell therapy. Additional safety evaluation for potential off-target gene editing,^108^ an undesired alteration to the genome, supported earlier observations^109^ with undetectable off-target editing and absence of sporadic oncogenesis during the follow-up period. No Cas9-specific immune reactions were detected, indicating that Cas9 immunogenicity is a marginal concern upon *ex vivo* SpCas9-RNP-engineered autologous cell product administration into immunosuppressed patients. Of note, the investigators did not disclose the duration of *ex vivo* cell product culture or the potential residual quantity of genome editor content in the final product.

Early clinical evidence on Cas9 immunogenicity and nuclease persistence in edited cell therapy products was reported in a phase 1 trial for neoantigen (i.e., cancer antigen) based TCR therapy for refractory cancers expressing the target antigen.^90^ Although the treatment did not render a long-term progression-free survival, the study provides valuable information on *ex vivo* multiplex Cas9-edited cell product safety through evaluation of the final product genomic integrity, genome editor persistence, and potential Cas9 immunogenicity. During an 11-day *ex vivo* manufacturing process, the target antigen (NY-ESO1 or LANE-1) and TCR were introduced to autologous T cells by lentiviral transfection. Additionally, to promote engraftment and cell persistence, three endogenous T cell function-related genes (*TRAC*, *TRBC*, and *PDC1*) were silenced with SpCas9-RNP cleavage-mediated gene knockout. In addition to on-target edits, the investigators detected chromosomal rearrangements and off-target indels in the T cells. The undesired changes did not result in a growth advantage, and gradually decreased during the *ex vivo* culture period and post-infusion monitoring. The genome editor’s persistence in the final cell products decreased below detection levels by day nine of *ex vivo* culture. No Cas9-specific humoral or cellular responses were observed post-infusion.

These two independent studies, supported by several others in autologous and allogenic applications,^107^^,^^110^^,^^111^^,^^112^ emphasize the immunological safety of *ex vivo* RNP-engineered cell products for anti-Cas9 host responses. Nonetheless, as the duration of antigen presentation plays a crucial role in host response regulation, the safety considerations for rapidly cleared RNP compared with intracellularly encoded nucleases are prone to vary. Furthermore, these studies underscore the importance of monitoring residual genome editor content during the manufacturing process, which has not been taken into consideration in early Cas9-based clinical trials,^113^^,^^114^ potentially resulting in genome editor carry-over to the recipients upon infusion.

#### Host response to *in vivo* Cas9-based genomic medicine

*In vivo* Cas9-based therapies, aiming to directly modify the function of genes within the affected tissues, are currently in various stages of preclinical and clinical development. The host immune response to Cas9 has emerged as a critical consideration, as the introduction of bacterial-derived Cas9 nuclease and associated nucleic acids into human tissues has been shown to activate innate and adaptive immune reactions.

##### Clinical observations of Cas9 immunogenicity

Therapeutic gene editing has progressed to late-stage clinical trials, demonstrating therapeutic benefit in monogenetic disorders. Notable examples include treatment approaches for rare genetic disorders, transthyretin amyloidosis (ATTR), a progressive and potentially fatal protein misfolding condition resulting in multi-organ dysfunction, and hereditary angioedema (HAE), a disease of recurrent and life-threatening edema attacks.

NTLA-2001 and NTLA-2002 are pioneering *in vivo* Cas9-based gene therapies targeting ATTR and HAE, respectively. Both utilize a similar approach: a single-dose LNP vector containing SpCas9 mRNA and sgRNA, for silencing of the disease-causing genes (*TTR*^115^ and *KLKB*^89^). The phase 1/2 trials for NTLA-2001 and NTLA-2002 demonstrated robust reduction in circulatory target protein levels (−90% serum TTR and over −67% plasma kallikrein, encoded by *TTR* and *KLKB*, respectively) resulting in improvement in disease symptoms. Both therapies were well tolerated, with no dose-limiting toxicities or severe adverse events (≥grade 3) reported. However, mild adverse events were observed, including a transient increase in serum liver transaminase content, peaking 1 day and 2 weeks after treatment infusion. Analysis of treatment component persistence revealed detectable plasma LNP and mRNA components several days after infusion, reducing to below detection levels 2 weeks and 1 month post-treatment, respectively. In both studies, regardless of the transient immunosuppression induced by glucocorticoids upon infusion, all patients developed anti-Cas9 antibodies, as detected by ELISA measurement, underlining Cas9 nuclease immune reactivity. Additionally, pre-existing Cas9 humoral immunity was observed in one of the study participants. Of note, the detected antibody response did not appear to affect treatment pharmacokinetics, pharmacodynamics, or safety. Nonetheless, observed increases in liver transaminases—indicative of liver damage—coincide with activation of potential cellular anti-Cas9 immune response. The lack of T cell-mediated response measurement in the study warrants further investigation. Following the positive results from phase 1/2, both treatments have advanced to phase 3 trials, MAGNITUDE (NCT06128629) and HAELO (NCT06634420) for NTLA-2001 and NTLA-2002, respectively. Together, these studies provided crucial insights into *in vivo* Cas9 host response and treatment outcome in humans. However, as LNP-mRNA-based delivery of Cas9 relies on intracellular nuclease expression, addressing MHC class I-mediated T cell immunity, potentially leading to cytolytic response, is of utmost importance and should be incorporated into the immunological safety assessment panels.

Additional insights into the immune responses to *in vivo* therapeutic editing are gained from the BRILLIANCE trial (NCT03872479) evaluating EDIT-101 in the treatment of Leber congenital amaurosis 10 (LCA10), a rare genetic eye disorder resulting in severe vision loss from birth or early childhood.^116^ A subretinal treatment with rAAV5 encoding for SaCas9 and sgRNA resulted in positive phase 1/2 trial results with lasting visual acuity improvement in a portion of the participants and an acceptable safety profile during a 2-year follow-up. Immunological assessments revealed pre-existing anti-AAV5 antibodies in 9/14 participants and anti-SaCas9 antibodies in 5/14 participants, with 2 participants maintaining SaCas9-specific antibodies post-treatment. Local inflammation and immune cell activation were observed, while novel humoral or cellular Cas9-specific host reactions were not detected in response to the treatment based on anti-Cas9 ELISA and circulatory PBMC ELISpot assays, respectively.

Highlighting the potential immunological risks associated with *in vivo* gene therapies, a recent N-of-1 clinical trial in Duchenne muscular dystrophy (DMD) resulted in a patient fatality.^80^ The study utilized an rAAV9 vector to deliver a CRISPR transactivator system containing a deactivated SaCas9 fused with VP64-transactivator unit (dSaCas9-VP64). Within 6 days of intravenous high-dose administration (1 × 10^14^ vg/kg), the patient experienced a severe cardiopulmonary toxic reaction, potentially due to an innate immune response to the high viral load. The acute reaction resulted in capillary leak syndrome with pericardial effusion on day 5, followed by acute respiratory distress syndrome (ARDS) on day 6, exacerbating the patient’s pre-existing right ventricular heart failure. Notably, ARDS is not typically associated with AAV gene therapies, suggesting a potential interplay between the therapy and the patient’s underlying condition. Postmortem analysis revealed minimal transgene expression in the liver, with undetectable levels in skeletal and cardiac tissues. In support of an acute innate-mediated reaction, treatment component-reactive antibodies or circulatory T cells were not detected. Considering the complex immunological challenges associated with *in vivo* gene therapies, understanding the potential immunogenic components of the treatment modality is crucial, particularly in patients with pre-existing inflammatory conditions.

In summary, emphasizing the potential of curative genomic medicine, early *in vivo* therapeutic gene editing trials have shown clinical benefit to patients with unmet medical needs. Moreover, they provide important insights into the immunological safety of CRISPR-Cas9 technology in humans.

##### Variables impacting Cas9 immunogenicity

The immunogenicity of Cas9 in CRISPR-based genomic therapies is influenced by a combination of variables, including the method of administration, disease conditions, and the intrinsic properties of Cas9. Understanding how these variables impact and potentially amplify Cas9 immunogenicity is central for the mitigation of unwanted immune reactions.

###### Host reactions in response to different administration approaches:

Host-specific factors play a crucial role in modulating Cas9 immunogenicity. These factors include the unique immune environment of the target tissues, pre-existing immunity, and individual genetic variability. The interplay among these factors can significantly influence the nature and magnitude of immune responses to Cas9-based therapies.

###### Cas9 immunity in immunoprivileged organs:

The host response to Cas9 in immune-privileged organs has been investigated in preclinical studies, revealing localized and systemic immune reactivity to the genome editing system. The eye is often regarded as an immunoprivileged organ due to its physical barriers, immunosuppressive signaling, and a marginal presence of APCs and effector immune cells.^117^ Cas9-based therapeutic gene editing has been successfully employed to target ocular diseases in preclinical studies involving mouse and nonhuman primate disease models of induced Leber congenital amaurosis 10 or macular degeneration.^118^^,^^119^^,^^120^ However, pro-inflammatory reactions, including local inflammation^120^ and Cas9-specific immune response, have been reported in response to rAAV vector-based intravitreal treatment in both mouse and nonhuman primate models,^119^ highlighting Cas9 immunoreactivity. Furthermore, low levels of anti-Cas9 antibodies have been detected in human vitreous fluid, with the antibody content correlating, albeit generally to a lesser extent, with serum levels. Moreover, the intravitreal anti-Cas9 antibodies increase drastically following intraocular *Streptococcus pyogenes* infection, highlighting the natural development of Cas9-immunity. In support of intravitreal antibody response, mice intramuscularly immunized with SpCas9 or ovalbumin present antigen-specific antibodies in the intravitreal fluid 6 weeks after the injection.^88^ In line with findings regarding the generation of anti-Cas9 immunity within immune-privileged organs, intracranial rAAV-SaCas9 or RNP-SpCas9 administration has been reported to result in a dose-dependent increase in immune cell tissue infiltration and Cas9-reactive splenocytes.^121^

Collectively, early evidence suggests that CRISPR-Cas9 genome editing in immune-privileged tissues is well tolerated; however, mild immunogenicity and the priming of the adaptive response can occur. Factors such as local inflammatory responses, prior infections, and disruptions of protective barriers during treatment administration influence host responses in these organs, necessitating further investigation into Cas9-related immune signaling in compartmentalized or so-called immune-privileged sites.

###### Anti-Cas9 immunity upon muscle tissue targeting:

Highly vascularized muscle tissue also features a unique immune environment characterized by active inflammatory signaling^122^ and antigen presentation via lymphatic tissue crosstalk.^123^ This feature is leveraged in vaccination^124^ to prime a protective antigen-specific immunity against pathogens; however, the reactive immune environment can also promote loss of tissue integrity in disease settings, such as inflammatory muscle diseases.^92^

In genetic muscular myopathies (e.g., DMD^92^) Cas9-based approaches are being studied as a therapeutic option. In animal models, these methods have demonstrated the ability to activate dystrophin expression and improve muscle function.^92^^,^^125^^,^^126^^,^^127^^,^^128^^,^^129^^,^^130^ In a DMD mouse model, a dual guide-LNP-SpCas9-mediated *in vivo* gene editing resulted in stable genomic exon skipping with approximately 10% indel generation, leading to improved contraction strength mediated by dystrophin expression. The exon skipping efficacy was observed to fluctuate and stabilize, with approximately 50% reduction from the peak efficacy, during a 12-month follow-up.^130^ Equivalent results have been reported in skeletal and cardiac muscles in mouse^131^ and canine^92^ models upon disease targeting with Cas9 and gRNA using a tissue-specific expression cassette to localize nuclease activity following intramuscular and systemic AAV vector delivery.

However, muscle-targeted treatment with Cas9 elicits innate and adaptive host responses,^132^ which can diminish the treatment benefit, especially upon viral vector delivery.^92^^,^^128^^,^^130^^,^^133^ Of note, significantly lower anti-Cas9 antibody levels have been reported upon LNP-Cas9 administration in comparison with AAV-mediated delivery,^130^ likely due to the shorter genome editor expression window and potentially lower immunogenicity of the vector system (i.e., adjuvant functionality of the vector system). Nonetheless, transient liver damage has been observed following limb perfusion treatment, which might be heightened upon systemic administration, as LNP vectors typically accumulate in the liver and lymphatic tissue.^123^ Moreover, the off-target tissue LNP accumulation can also reduce the treatment biodistribution to the target tissue.

In the muscle, Cas9 expression can promote target cell rejection. For example, rAAV-mediated Cas9 delivery to both non-inflamed and inflamed (DMD disease model) muscle can generate a dose-dependent humoral and cellular response.^92^ Furthermore, a substantial reduction in Cas9 expression combined with increased CD4+ and CD8+ T cell infiltration, and elevated muscle cytokines indicates a rejection of edited cells, potentially due to Cas9 or the genome editing product recognition as non-self. In contrast, in the same study, animals treated with SERCA2a- and micro-dystrophin encoding vectors (control vectors) show transgene tolerance with minimal T cell infiltration and pro-inflammatory cytokine expression, along with the presence of FoxP3+ regulatory T cells. Notably, the anti-Cas9 host response and loss of edited cells were reported to occur upon transient prednisolone immunosuppression, potentially due to transgene expression exceeding the immunosuppression window.

Together, these observations provide indirect evidence on immune-mediated rejection of Cas9-expressing cells, emphasizing the need to reduce prolonged genome editor expression and epitope presentation in the treated cells. Moreover, further studies are needed to develop effective immunosuppression strategies in Cas9-based genomic medicine.

###### Host response to systemic genome editor administration:

Systemic infusion is a therapy administration route commonly used in gene therapy for a broad distribution of the therapeutic agent.^115^^,^^134^^,^^135^^,^^136^ The administered doses required for effective treatment are typically high with the therapeutic modalities accumulating in the liver, and an increased risk of acute treatment-induced adverse events, including immune-mediated reactions,^135^ when compared with localized treatments. Currently, Cas9-based *in vivo* gene targeting with systemic administration is studied for therapeutic potential in various genetic diseases.^115^^,^^128^^,^^137^^,^^138^^,^^139^^,^^140^

One of the pioneering reports on Cas9 immunogenicity following systemic administration is based on the generation of a preclinical liver disease model for *phosphatase and tensin homolog* (*PTEN*) dysfunction-driven nonalcoholic steatohepatitis.^136^ To impair PTEN function in the liver, SpCas9- and *PTEN*-targeting sgRNA were delivered to the target tissue with an adenoviral (Ad) vector. The resulting indel formation in the target gene, along with the development of symptoms characteristic of the desired disease model (e.g., lipid accumulation and liver enlargement), shows successful genome editing and disease model generation. Nevertheless, in addition to the successful editing outcome, animals presented SpCas9-specific humoral and cellular immune responses, together with vector-induced toxicity typically observed with high-dose Ad vector administration. Similarly, Cas9-specific T cell responses upon systemic administration have been reported in a therapeutic context with Cas9 naive animals treated with other genome editor modalities, such as rAAV-SaCas9,^128^ rAAV-SpCas9, or LNP-SpCas9.^140^ Moreover, upon secondary exposure in pre-immunized mice, Cas9 administration results in rapid anti-Cas9 response^140^ and diminishes treatment outcome,^128^ highlighting the importance of Cas9-specific T cell immunity screening, especially when the genome editor is delivered with viral vectors that have prolonged intracellular cargo expression. Intriguingly, systemic delivery of LNP-encapsulated RNP^139^—aiming to reduce systemic Cas9 exposure and nuclease persistence in tissues—resulted in therapeutically relevant editing outcomes in preclinical models of familial hypercholesterolemia and childhood interstitial lung disease-related genes, *PCSK9* and *SFTPC*, respectively. Based on a host immune response evaluation during a limited 2-week follow-up period, the novel delivery approach was well tolerated in animal models, without acute elevation in pro-inflammatory cytokines or liver transaminases, in support of previous reports with LNP-SpCas9 mRNA infusions.^138^

Taken together, observations from preclinical studies highlight Cas9 immunoreactivity, which, depending on the therapeutic context, can influence treatment outcomes. The host immune response to a genome editor is variable, stressing the importance to evaluate potential additive immunogenicity of treatment and host-related parameters.

###### Delivery vector system—A potential adjuvant for Cas9 immunogenicity:

For the delivery of genome editors, numerous viral and non-viral options are available with their benefits and drawbacks.^137^^,^^141^^,^^142^ Key factors for the optimal delivery approach include: (1) the vector systems' capacity to package the genome editor, (2) the ability of the vector system to transport the cargo to the target tissue while minimizing off-target delivery, (3) transient cargo expression, and (4) low vector-mediated immunogenicity.

**Figure 3:**
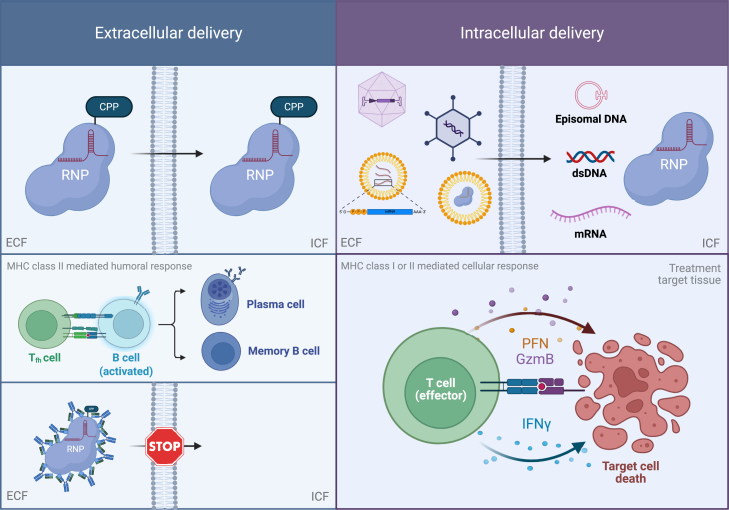
Different genome editor delivery strategies can impact the host response Extracellular delivery formats such as CPP-tagged RNP engage MHC class II pathways, promoting B cell activation and antibody production. Pre-existent or treatment-induced anti-Cas9 antibodies can impact treatment efficacy and drug pharmacokinetics. In contrast, intracellular delivery (e.g., viral or nanoparticle) of Cas9 drives cytotoxic T cell responses, potentially resulting in target cell rejection and tissue damage. CPP, cell-penetrating peptide; dsDNA, double-stranded DNA; ECF, extracellular fluid; GzmB, Granzyme B; ICF, intracellular fluid; IFN-γ, interferon-gamma; mRNA, messenger RNA; PFN, perforin; Tfh, T follicular helper cells.

Several viral vector systems, relying on cargo delivery to cells as DNA, are used for genome editor delivery. For these approaches, Ad vectors have a long history in gene therapy. Good tissue transduction and cargo (dsDNA) packaging capacity present these vectors as an option for the delivery of large genome editors together with gRNA, especially for challenging gene therapy target tissues such as muscle. However, a high vector immunogenicity^78^^,^^143^ and an extended cargo expression window remain significant challenges for Ad vector-mediated delivery,^142^ and can potentiate Cas9-specific host responses. Currently, rAAV vectors are the most widely utilized viral vector systems in gene therapies due to their low immune reactivity in humans and diverse serotype tissue tropism for selective tissue delivery. Nevertheless, for genome editor delivery, the utility of rAAV vectors is restricted by their limited cargo (ssDNA) packaging capacity and the risk of cargo integration into the host genome. Moreover, similarly to other viral vectors, AAV capsid immunogenicity combined with a prolonged cargo expression window is an important safety consideration.^137^^,^^141^^,^^142^ Alternative viral vector systems (e.g., lipid-membrane enveloped non-integrating retroviral vectors, and viral-like particles^144^) are gaining interest for targeted delivery, but also present an immune-related safety concern. Importantly, persistent intracellular transgene expression^145^^,^^146^ increases the risk of cargo (i.e., Cas9) specific cellular response against the host tissue (Figure 3).

Nanoparticle delivery systems (e.g., LNPs) provide an alternative approach to viral vectors and have emerged as a promising solution for delivering genome editors. LNPs, which typically transport the therapeutic agent in mRNA format, have demonstrated good tolerability and therapeutic benefit in humans,^115^ indicating their immunological tolerability. Although generally considered less immunogenic to most viral vectors, LNP administration can provoke both innate and adaptive host responses,^89^^,^^115^ with the LNP-mediated immunogenicity being largely variable based on their lipid composition and formulation.^147^ The intracellular presence of LNP-delivered cargo (e.g., mRNA or recombinant protein) typically decreased over a 24-h period.^138^ However, recent in-human observations^89^^,^^115^ indicate a potential for systemic persistence of LNP particles and their cargo over several days post-infusion, presenting a concern for class I and II mediated immunogenicity.

Additional delivery systems such as gold^148^ and polymeric nanoparticles^149^ are explored for nucleic acid delivery, offering advantages in terms of size control, surface functionalization, and the potential for tissue-specific targeting.^150^ However, similarly to the LNPs, the immunogenicity profile of these nanoparticle systems varies depending on the particles’ size and surface modifications.^151^^,^^152^ Nonetheless, the diversity of nanoparticle systems offers opportunities for tailored delivery strategies, and consideration of their immunogenic potential when combined with Cas9 administration requires further attention.

Moreover, cell-penetrating peptides (CPPs)^150^^,^^153^^,^^154^ have emerged as promising alternatives to viral and nanoparticle-based delivery systems for CRISPR-Cas9 components. These approaches include CPP-tagged delivery of unencapsulated nuclease or RNP,^153^^,^^155^ and CPPs encapsulated genome editors in various formats, providing an approach for selective cellular uptake and nuclease turnover.^156^ However, CPPs may contain immunostimulatory epitopes or endotoxin contaminants from the CPP or protein production pipeline,^121^ that may potentiate pro-inflammatory host response. In addition to the potential class I-mediated response against CPP or the cargo, infusion of CPP-tagged protein (e.g., Cas9) carries concern for class II-driven humoral response (Figure 3).

For clinical considerations, selecting an optimal vector system with minimal intrinsic immunogenicity is crucial. However, given the multitude of delivery method options and their varying characteristics, this selection is not trivial. Depending on the delivery method, the immune-related clinical consequences differ; the humoral response may limit dosing, while the cytotoxic class I- or II-mediated response presents an immunotoxicity concern, especially when targeting critical organs (e.g., liver) (Figure 3). Thus, careful consideration is essential when selecting the genome editor delivery method.

###### Genome editor-associated nucleic acids as amplifiers of Cas9 immunogenicity:

Upon viral and nanoparticle delivery, the genome editor is introduced to the cells in the molecular form of RNP or nucleic acid. As part of maintaining cellular integrity, innate sensing of endo- and exogenous nucleic acids via PPRs activates DAMP and PAMP signaling cascades, leading to the secretion of pro-inflammatory cytokines and chemokines and subsequent activation of innate and adaptive immune cells.^36^^,^^157^ For viral vectors, successful genome editing requires endosomal escape and intracellular release of the nucleic acid cargo. In the endosomes or within the cytoplasm, exogenous dsDNA and ssDNA from Ad or AAV vectors, respectively, are recognized by DNA sensors (e.g., TLR9), resulting in activation of various pro-inflammatory signaling cascades, including AIM2-mediated cytokine maturation and activation of cGAS-STING-pathway.^36^^,^^157^ Similarly, exogenous dsRNA or ssRNA can activate cytoplasmic RNA sensors (e.g., RIG-I^158^^,^^159^ or TLR7/8^160^) and consecutive pro-inflammatory signaling cascades, as observed through the development of RNA-based therapeutics.^161^ Independent of the delivery approach, the pro-inflammatory innate response to vector cargo can promote a local inflammatory tissue environment and serve as an amplifier of the Cas9-specific host response.

##### Intrinsic pro-inflammatory features of Cas9

Finally, in addition to genome editor-related nucleic acids, the intrinsic characteristics of the nucleases contribute to the development of Cas9-specific immunity. As stated, prokaryotic Cas9 contains immunostimulatory epitopes that trigger the priming and activation of adaptive immune responses through the pMHC complex binding and TCR recognition. These epitopes vary in amino acid composition and length based on the MHC class and allele. For Cas9, the current understanding of immunodominant epitopes is limited to SpCas9^99^ and SaCas9,^101^ focusing on HLA-A and HLA-DBR1 for class I and II, respectively. Interestingly, for a portion of the identified SpCas9 epitopes, sequence homology can be observed across various Cas9 orthologs, while some exhibit HLA restriction. Furthermore, although to a lesser degree, some epitopes share sequence similarity with non-Cas9 bacterial proteins.^99^ These studies present the first evidence of Cas9 class I and II immunodominant epitopes in humans, as well as on potential epitope cross-reactivity, especially for short class I epitopes. Notably, the identification of immunodominant epitopes can be utilized for screening Cas9-specific T cell responses, which is particularly important for class I epitopes for recognizing potential cytolytic cellular responses. However, given the limited understanding of Cas9 epitopes, peptide-based host response detection remains challenging and prone to false negative errors due to HLA polymorphism and the sheer number of potential epitopes.

Additionally, the enzymatic activity of the nuclease induces genotoxic stress and potentially promotes a pro-inflammatory response. DNA cleavage and DDR activation are a requirement for successful genome editing but can also stimulate DNA damage-induced inflammatory signaling in cells experiencing genotoxic stress.^162^^,^^163^ DNA cleavage detection activates various intermediate DDR enzymes, such as ATM- and ATR-protein kinases. In addition to regulating DDR processes, these kinases upregulate immunostimulatory proteins such as NKG2D receptor ligands.^162^^,^^163^ NKG2D receptor is a costimulatory membrane protein expressed by cytolytic immune cells, which upon ligand binding positively regulates effector cell activity.^164^ (Figure 2). Moreover, DAMP-mediated inflammatory responses, including the DSB-induced cGAS-STING-pathway^165^ and SSB-mediated NF-κB activation^166^ promote cytokine production and release by the damaged cell. These diverse signaling pathways collectively contribute to the inflammatory response following DNA cleavage, adding a new layer to the importance of considering pro-inflammatory processes when developing genome-targeted therapies.

Overall, several host-specific and genome editor-related factors contribute to the development of treatment-induced immunity to Cas9. Some of these factors, such as vector selection-related immunogenicity, can be addressed by choosing alternative approaches that are suitable for the specific aim. In contrast, DNA cleavage-induced inflammatory responses might only be addressable through pharmacological interventions.

## Strategies to mitigate Cas9 immunogenicity

In the era of *in vivo* gene editing, several strategies—each with its own complementary benefits—are being investigated to reduce Cas9 immunogenicity. These approaches include administering treatment alongside traditional immunosuppression regimens, limiting systemic nuclease persistence and rational reduction of nucleic acid or nuclease immunogenicity.

Upon *in vivo* delivery, transient corticoid-based immunosuppression is employed to mitigate treatment-induced immunogenicity. However, short-term immunosuppression regimens fail to prevent the activation of an adaptive host response upon AAV^92^ or LNP delivery,^89^^,^^115^ indicating that revised strategies are needed.

To minimize cellular Cas9 persistence and the immunogenicity of exogenous nucleic acid, direct RNP delivery and improved production protocols for endotoxin removal have been implemented to address Cas9-specific responses.^121^ Similarly, directed self-cleavage of Cas9 nucleases could be utilized to reduce long-term expression of the nuclease upon viral delivery.^167^ Additionally, to mitigate potential anti-Cas9 humoral response and off-tissue delivery upon administration, RNP encapsulation to tissue-targeted LNPs has been presented as a delivery approach.^139^ Alternatively, to circumvent pre-existing immunity or to enable recurrent dosing, Cas9 nucleases without shared epitopes could be a way to circumvent adaptive memory responses.^168^ Furthermore, numerous approaches to mitigate delivery vector immunogenicity,^168^^,^^169^ such as viral capsid masking,^157^ could be used to address genome editor delivery-related immunogenicity.

To mitigate the exogenous nucleic acid-induced pro-inflammatory innate response, a reduction of unmethylated CpG-islet content in the sequence can be used during both Cas9 and gRNA design to reduce the TLR9 activation.^170^ Further chemical modifications, such as 2′-O-methyl or phosphorothioate linkages, can reduce TLR7/8 recognition and innate immune activation to chemically synthesized gRNAs. Also, the removal of 5′-triphosphate groups from *in-vitro*-transcribed sgRNAs is an approach to decrease the immunogenicity of synthetic nucleic acids.^171^^,^^172^

Moreover, rational epitope engineering has emerged as a promising approach to reduce Cas9 variant immunogenicity.^98^^,^^99^^,^^101^ Several diverse methods, such as phage library mapping,^132^ *in silico* prediction of antigenic epitopes,^99^ or functional immunopeptidome analysis of MHC class I and II presented epitopes, can be used for antigenic peptide identification.^98^^,^^99^^,^^101^ However, these approaches are challenged by the vast HLA-haplotype diversity, and collaborative efforts will be required for comprehensive immunodominant epitope mapping. Notably, engineering potential epitopes can reduce peptide immunogenicity.^98^^,^^99^ However, this approach is limited to regions that do not compromise the nucleases’ enzymatic activity. Furthermore, the immunomodulator benefit of epitope engineering is specific to the HLA allele of interest, presenting a potential need for nuclease customization for patient-specific haplotypes. Nevertheless, epitope-engineered Cas9-peptides have demonstrated reduced T cell activation *in vitro* and *in vivo* in preclinical models, paving the way for nuclease engineering for host response mitigation.

## Future perspectives and challenges

Moving forward, addressing Cas9 immunogenicity presents both exciting opportunities and significant challenges. The development of next-generation Cas9 nucleases, engineered for reduced immunogenicity while maintaining or enhancing editing efficiency, remains a key focus. Advances in computational biology and machine learning may facilitate more accurate predictions of immunogenic epitopes across diverse HLA types, enabling the design of HLA-relaxed Cas9 variants to mitigate nuclease immunogenicity. However, challenges persist in balancing the nuclease immunogenicity mitigation with preserved functionality, as well as in addressing the variability of immune responses across different tissues and delivery methods. The long-term effects of pre-existing Cas9 immunity and repeated nuclease administration, particularly in the context of potential memory T cell responses, require further investigation. Additionally, the development of sensitive and standardized assays for detecting and quantifying anti-Cas9 immune responses in clinical settings is crucial for accurate assessment of the treatment safety and efficacy. Hence, further efforts such as Cas9 variant sequence similarity comparisons with immunogenic proteins or alternative Cas family nucleases would benefit the evaluation of potential Cas9 cross-reactivity upon assay development. Importantly, as the field of therapeutic genome editing advances, similar questions regarding intrinsic nuclease immunogenicity^98^ are relevant for other Cas family enzymes^173^^,^^174^ and their engineered variants. Intriguingly, observations of pre-existing Cas9-specific regulatory T cell populations^100^ suggested the need to further explore the impact of Cas9 tolerance on genome editing outcomes, as well as potential strategies for inducing tolerance to Cas9 or the newly generated variants of the dysfunctional target protein.^74^^,^^75^ Ultimately, addressing Cas9 immunogenicity will likely require a multifaceted approach, combining optimized protein design, delivery strategies, and novel methods to regulate intracellular persistence of Cas9 for the realization of the full therapeutic potential of the technology.

## Conclusions

The immunogenicity of Cas9 presents a complex challenge in the clinical application of CRISPR-based genomic medicines. The interaction between delivery vectors, nuclease properties, and host immune responses necessitates a multilayered approach to mitigating immune reactions. Current clinical reports highlight the curative potential of therapeutic genome editing, but also underscore the risk of activating the adaptive immune response, even upon transient immune suppression and minimized nuclease half-life. Progress in mitigating adaptive responses has been made through the development of less immunogenic Cas9 variants by rational epitope engineering, optimization of the delivery systems to reduce systemic exposure, and modification of gRNAs to minimize innate immune activation. These advances, combined with improved immunosuppression strategies, present promising avenues for *in vivo* Cas9-based therapies. However, the diversity of human immune responses and the potential for long-term adaptive immunity against Cas9 warrants the need for continued research and personalized approaches. As the field moves forward, integrating immunological considerations into the design of CRISPR-Cas9 therapies from the outset will be crucial. Additionally, the measurement of immunological responses will likely rely on a combinatorial approach that incorporate computational and functional readouts. Hence, efforts to develop and harmonize sensitive screening methodology for host immune response against CRISPR-Cas9-based therapeutics are of high importance. The ultimate goal remains to harness the full potential of this technology for diseases with unmet needs, while ensuring its safety and efficacy across diverse patient populations and therapeutic applications.

## Acknowledgments

This project has received funding from the European Union’s Horizon Europe research and innovation program under the 10.13039/100010665Marie Skłodowska-Curie Actions grant agreement no. 101072427 (GetRadi – Gene Therapy of Rare Diseases). Views and opinions expressed are, however, those of the authors only and do not necessarily reflect those of the European Union or the European Research Executive Agency. Neither the European Union nor the European Research Executive Agency can be held responsible for them. We thank Prof. Vincenzo Cerullo and the members of IVT lab for their valuable support and guidance. Figures were created with BioRender.com.

## Author contributions

All authors co-wrote, edited, and approved the manuscript. V.S. assembled the figures.

## Declaration of interests

All authors are employees and shareholders of AstraZeneca. V.S. is a Marie Skłodowska-Curie Actions Doctoral Network PhD student at AstraZeneca. A.L.C. is the inventor on the FaDe-Cas9 patent (WO2020049158A1) that, although not mentioned in this review, has application on Cas9 immunogenicity reduction.
